# Spinal V3 Interneurons and Left–Right Coordination in Mammalian Locomotion

**DOI:** 10.3389/fncel.2019.00516

**Published:** 2019-11-20

**Authors:** Simon M. Danner, Han Zhang, Natalia A. Shevtsova, Joanna Borowska-Fielding, Dylan Deska-Gauthier, Ilya A. Rybak, Ying Zhang

**Affiliations:** ^1^Department of Neurobiology and Anatomy, College of Medicine, Drexel University, Philadelphia, PA, United States; ^2^Department of Medical Neuroscience, Brain Repair Centre, Faculty of Medicine, Dalhousie University, Halifax, NS, Canada

**Keywords:** spinal cord, central pattern generator, locomotion, commissural neurons, V3, optogenetic stimulation, computational modeling

## Abstract

Commissural interneurons (CINs) mediate interactions between rhythm-generating locomotor circuits located on each side of the spinal cord and are necessary for left-right limb coordination during locomotion. While glutamatergic V3 CINs have been implicated in left-right coordination, their functional connectivity remains elusive. Here, we addressed this issue by combining experimental and modeling approaches. We employed Sim1^Cre/+^; Ai32 mice, in which light-activated Channelrhodopsin-2 was selectively expressed in V3 interneurons. Fictive locomotor activity was evoked by NMDA and 5-HT in the isolated neonatal lumbar spinal cord. Flexor and extensor activities were recorded from left and right L2 and L5 ventral roots, respectively. Bilateral photoactivation of V3 interneurons increased the duration of extensor bursts resulting in a slowed down on-going rhythm. At high light intensities, extensor activity could become sustained. When light stimulation was shifted toward one side of the cord, the duration of extensor bursts still increased on both sides, but these changes were more pronounced on the contralateral side than on the ipsilateral side. Additional bursts appeared on the ipsilateral side not seen on the contralateral side. Further increase of the stimulation could suppress the contralateral oscillations by switching to a sustained extensor activity, while the ipsilateral rhythmic activity remained. To delineate the function of V3 interneurons and their connectivity, we developed a computational model of the spinal circuits consisting of two (left and right) rhythm generators (RGs) interacting via V0_V_, V0_D_, and V3 CINs. Both types of V0 CINs provided mutual inhibition between the left and right flexor RG centers and promoted left-right alternation. V3 CINs mediated mutual excitation between the left and right extensor RG centers. These interactions allowed the model to reproduce our current experimental data, while being consistent with previous data concerning the role of V0_V_ and V0_D_ CINs in securing left–right alternation and the changes in left–right coordination following their selective removal. We suggest that V3 CINs provide mutual excitation between the spinal neurons involved in the control of left and right extensor activity, which may promote left-right synchronization during locomotion.

## Introduction

The rhythmic activities controlling locomotor movements in mammals are generated by neural circuits within the spinal cord, representing so-called central pattern generators (CPGs; Graham Brown, 1911; Grillner, 1981, 2006; Kiehn, 2006, 2011; Rossignol et al., 2006). It is commonly accepted that each limb is controlled by a separate spinal CPG and that CPGs controlling fore and hind limbs are located in the left and right sides of cervical and lumbar enlargements, respectively. These CPGs are connected by spinal commissural interneurons (CINs) that coordinate their activities, hence defining locomotor gaits. CINs project axons across the spinal cord midline and affect interneurons and motoneurons located on the contralateral side of the cord (Kjaerulff and Kiehn, 1997; Butt and Kiehn, 2003; Quinlan and Kiehn, 2007; Jankowska, 2008).

Several classes of spinal CINs, including two subtypes of V0 CINs (excitatory V0_V_ and inhibitory V0_D_) and the excitatory V3 CIN, have been identified based on their transcription factor profiles (Lanuza et al., 2004; Goulding, 2009; Gosgnach, 2011). Both subtypes of V0 CINs promote left–right alternation, and their functional ablation results in aberrant left–right synchronization. *In vitro*, selective ablation of V0_D_ CINs disrupted left-right alternation at low locomotor frequencies while ablation of V0_V_ CINs disrupted left-right alternation at higher locomotor frequencies (Talpalar et al., 2013). Thus, V0 CINs are necessary for securing left-right alternation.

*In vivo*, an increase of locomotor speed in rodents is accompanied by a transition from left–right alternating gaits (walk and trot) to left-right synchronized gaits (like gallop and bound). Yet, the CIN networks promoting left–right synchronization at higher locomotor speeds, or during V0 ablation, are poorly understood. Our previous modeling studies suggested that left-right synchronization could be performed by V3 CINs providing mutual excitation between the left and right rhythm-generating circuits (Rybak et al., 2013, 2015; Shevtsova et al., 2015; Danner et al., 2016, 2017; Shevtsova and Rybak, 2016). Although this suggestion allowed our previous models to reproduce the results of the above experimental studies, including the effects of V0 CIN ablation *in vitro* and *in vivo*, the role and connectivity of V3 neurons suggested by these models have not been tested experimentally and remained hypothetical.

V3 interneurons, defined by their post-mitotic expression of the transcription factor single-minded homolog 1 (Sim1), are excitatory, and the majority of them project to the contralateral side of the spinal cord (Zhang et al., 2008). Genetic deletion of V3 interneurons did not affect left-right alternation, but caused unstable gaits in walking mice, and generated imbalanced and less robust rhythmic fictive locomotion in isolated neonatal spinal cords (Zhang et al., 2008). While these experimental data strongly suggest that V3 interneurons are involved in the control of locomotion, their exact function and commissural connectivity remain mainly unknown.

To address V3’s functional connectivity between left-right spinal circuits, we used *in vitro* preparations of isolated spinal cords from neonatal mice, in which fictive locomotion was induced by neuroactive drugs. This preparation allows studying functional connectivity between genetically identified spinal interneurons, involved in CPG operation and left-right coordination. We took advantage of an optogenetic approach, which enabled us to specifically regulate the activity of V3 interneurons on each side of the isolated spinal cord during fictive locomotion. We then designed an updated computational model of spinal circuits that incorporated the connectivity of V3 CINs suggested from our experimental studies. Together our experimental and modeling results provide convincing evidence that V3 interneurons contribute to synchronization of the left-right locomotor activity (under appropriate conditions) by providing mutual excitation between the extensor centers of the left and right CPGs.

## Results

### Optical Activation of Lumbar V3 Interneurons Increases the Intensity of Extensor Motor Activity and Slows Oscillation Frequency of Drug-Evoked Fictive Locomotion

To assess the function of V3 interneurons in the spinal locomotor network, we used an optogenetic approach that allowed us to selectively activate V3 interneurons in different regions of the isolated spinal cords from *Sim1^Cre/+^*; *Rosa26 ChR2-EYFP* (Sim1cre-Ai32) mice, which express channelrhodopsin2 (ChR2) and enhanced yellow fluorescent protein (EYFP) in Sim1 positive cells.

**FIGURE 1:**
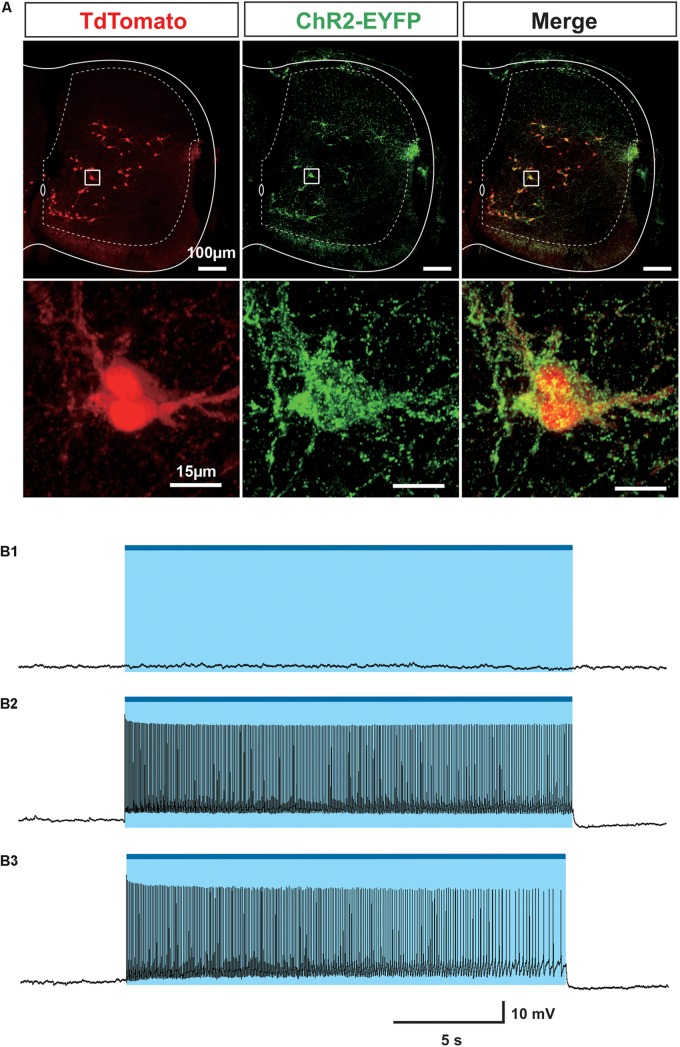
Investigation of Sim1 cell in Sim1^Cre/+^; Ai32 mouse. **(A)** ChR2-EYFP (Green) and tdTom (red) co-express in Sim1 + V3 interneurons in the spinal cord of Sim1^Cre/+^; tdTom; Ai32 mice. Upper: representative image of a cross section of a spinal cord from Sim1^Cre/+^; tdTom; Ai32 mouse. Lower: enlarged image of the insets. **(B1)** Light did not activate EYFP negative cell. **(B2)** Patch clamp recordings of Sim1^Cre/+^; Ai32 cell with optical activation. **(B3)** Patch clamp recording of Sim1^Cre/+^; Ai32 cell with optical activation with fast synaptic transmission blocker. Blue bar and shaded blue area represent the optical activation (light-on) period.

To verify the expression of ChR2-EYFP in Sim1 positive V3 interneurons, we crossed Sim1cre-Ai32 with *Rosa26tdTom (Ai14)* to generate Sim1^Cre/+^; tdTom; Ai32 mice. Sim1^Cre/+^; tdTom has been well characterized and widely used in our previous studies (Borowska et al., 2013, 2015; Blacklaws et al., 2015). In Sim1^Cre/+^; tdTom; Ai32 spinal cords, ChR2-EYFP fusion protein could be specifically detected around all tdTom positive cells (Figure 1A), which demonstrated the co-expression of ChR2-EYFP and tdTom in Sim1+V3 interneurons. Using whole-cell patch-clamp recordings, we confirmed that the blue fluorescent light (488 nm) could produce membrane depolarization and evoke persistent spiking only in EYFP expressing cells (22/22) from the slices of Sim1cre-Ai32 or Sim1^Cre/+^; tdTom; Ai32 mice at postnatal day (P) 2–3 (Figures 1B1–B3). None of EYFP negative cells (10/10) showed any direct response to the light (Figure 1B1). The evoked spiking activity continued within a 20-s period with or without glutamatergic receptor blockers (CNQX and AP-5; Figures 1B2,B3). These results confirmed that V3 interneurons in the isolated spinal cords of Sim1cre-Ai32 mouse could be selectively activated by the blue fluorescent light.

**FIGURE 2:**
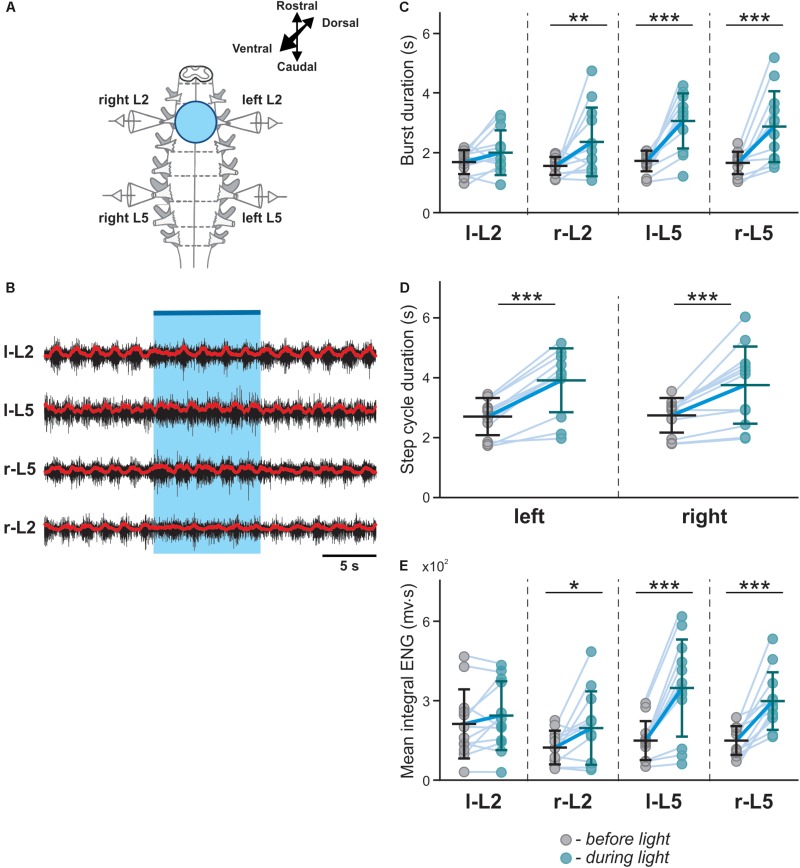
Symmetric optical activation of both sides of V3 interneurons in L2 ventral region during drug-evoked fictive locomotion and changes in ENG characteristics. **(A)** Experimental setup. **(B)** Optical activation of lumbar V3 interneurons increases the intensity of extensor motor output and slows oscillation frequency of drug-evoked fictive locomotion. Fictive locomotion was induced by drug cocktail (5-HT 8 μM, NMDA 8 μM) in Sim1cre-Ai32 cord. The blue bar on the top and shaded blue area represent the optical stimulation period. Red lines are rectified and smoothed traces of the original ENG recordings (black). **(C–E)** Burst duration, step cycle duration, and mean integral ENG during drug-induced locomotor-like activity (*n* = 12) before (gray circles) and during (blue circles) optical activation. Each circle represents the average value of estimated parameter for one trace. Light blue lines connecting two circles represent parameter increase in one cord. Thick, dark blue line represent the average of increase. ^∗^ indicates *P* < 0.05, ^∗∗^ indicates 0.01 < *P* < 0.05, ^∗∗∗^ indicates 0.0001 < *P* < 0.001.

To investigate the role of V3 interneurons and their interactions with CPG circuits, we shined fluorescent light onto the ventral spinal cord of neonatal Sim1cre-Ai32 mice during fictive locomotion evoked by a 5-HT/NMDA mixture (5-HT 8 μM, NMDA 7–8 μM) and analyzed the effects of light stimulation on the ongoing rhythmic activity (Figure 2). Flexor and extensor activities on each side of the cord were evaluated based on the recordings from L2 and L5 ventral roots, respectively. Optogenetic stimulation with blue fluorescent light applied on the whole L2 ventral spinal cord (Figure 2A) slowed down the ongoing rhythmic activity, as evident by an increased locomotor cycle period (Figures 2B,D; *n* = 12, left L5 *P* = 0.0005, right L5 *P* = 0.0010). The increased cycle duration was mainly attributed to an increase in the L5-burst durations, while L2-burst durations did not significantly change in response to the applied stimulation (Figures 2B,C). Furthermore, the optogenetic stimulation caused an increase in the amplitude of integral ENG bursts in L5, but not in L2 ventral roots (Figures 2B,E). These results suggest that lumbar V3 neurons interact with locomotor CPGs and provide activation of extensor circuits, either directly or transynaptically.

### Biased Optical Activation of V3 Interneurons in Spinal Segment L2 Leads to Asymmetrical Left–Right Motor Activity

**FIGURE 3:**
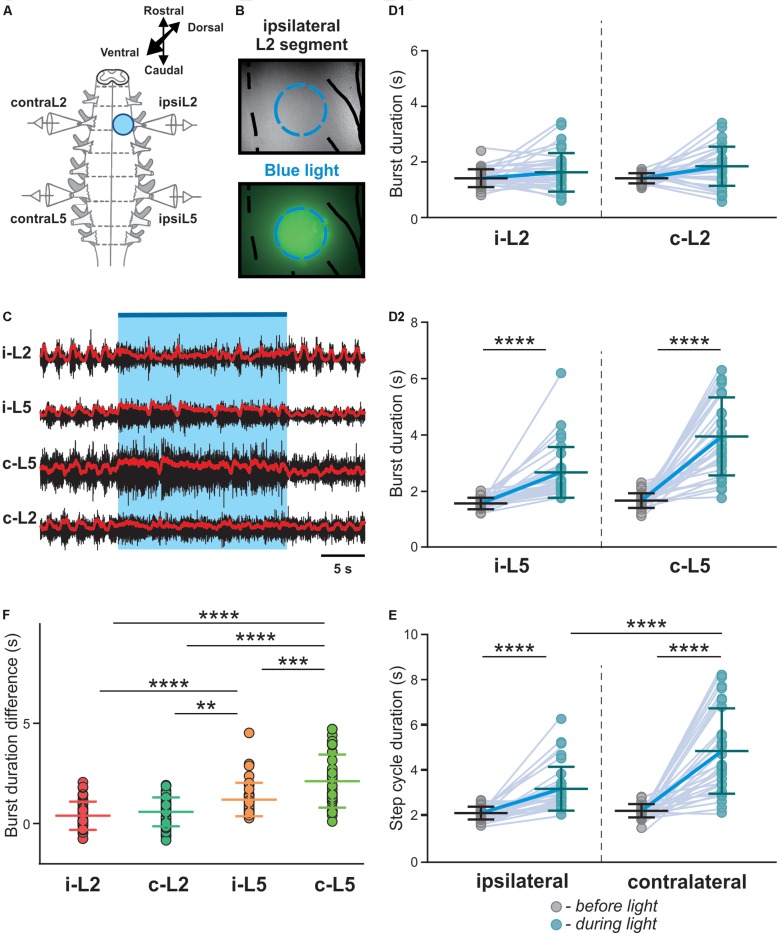
Biased optical activation of V3 interneurons in spinal segment L2 and corresponding changes in ENG characteristics in spinal segment L2 and L5 during drug-induced locomotor-like activity. **(A)** Illustration of experimental setup for recording motor activity evoked by optical stimulation on the left spinal segment L2 from ventral side during drug-evoked fictive locomotion. **(B)** Image of the optical stimulation area. Black dashed line represents the midline of spinal cord. Blue dashed circle illustrates the light illuminated region. Black line on the right shows the frame of Nerve L2 and lateral edge of the spinal cord. **(C)** Recording trace during optical stimulation. Biased optical activation of V3 interneurons in spinal segment L2 leads to asymmetrical left-right extensor motor activity. Blue bar and area indicate the stimulation period. Red lines are rectified and smoothed traces of the original ENG recordings (black). **(D1,D2)** Average of burst duration of L2 **(D1)** and L5 **(D2)** during drug-induced locomotor-like activity (*n* = 33) before (gray circles) and during (blue circles) optical stimulation in ipsilateral and contralateral cord. **(E)** The average of step cycle duration of ipsilateral and contralateral L5 activities during drug-induced locomotor-like activity (*n* = 33) before (gray circles) and during (blue circles) optical stimulation. Light blue lines connecting two circles represent change in one cord. Thick, dark blue line represent the average of change. ^****^ indicates *P* < 0.0001. **(F)** Average of locomotor burst duration difference between before and during biased optical stimulation. ^∗∗^ indicates 0.001 < *P* < 0.01, ^∗∗∗^ indicates 0.0001 < *P* < 0.001, ^****^ indicates *P* < 0.0001; *n* = 33.

Since most V3 neurons are commissural interneurons, activation of V3s on one side of the spinal cord should more strongly impact the contralateral circuits. To test this hypothesis, we used a 20x, 1.0 numerical aperture (NA) objective to deliver the light onto a small area on one side of the spinal cord (Figure 3A). We then selected the illuminated region by manually adjusting the field diaphragm to have the activation zone between approximately one-third to a half of the spinal cord (Figure 3B) and the intensity of the light-emitting diode (LED) light to regulate the number of V3 neurons being activated.

Under these experimental conditions, we found that optical activation of V3 interneurons on one side of the cord significantly prolonged L5 burst durations and step cycles on both sides (Figures 3C,D2,E; *P* < 0.0001), while L2 burst durations were not significantly affected (Figure 3D1). However, the contralateral L5 burst durations were influenced more strongly than those of ipsilateral L5 (Figure 3F). Consequently, the step-cycle period also changed more strongly on the contralateral side than the ipsilateral side (Figure 3E), which led to a left-right asymmetric activity with more bursts on the ipsilateral than on the contralateral side. Emergence of additional bursts in an integer relationship is a fundamental property of (weakly-) coupled oscillators, with asymmetric drive (Pikovsky et al., 2001; Pikovsky and Rosenblum, 2003; Rubin et al., 2011), suggesting that tonically activated V3 neurons mainly affect (and slow down) the contralateral CPG.

**FIGURE 4:**
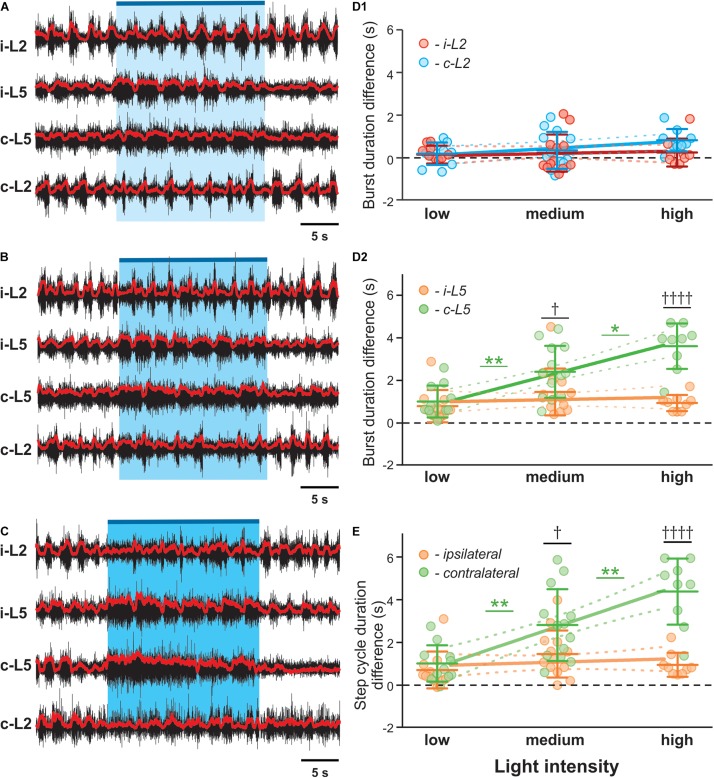
V3 activated contralateral extensor motor activity is correlated with optical stimulation intensity. Representative traces of locomotor activity induced by drug (5-HT 8 μM, NMDA 8 μM) in Sim1^Cre/+^; Ai32 cord at low **(A)**, medium **(B)**, and high **(C)** light intensity. Blue bar on the top of each trace and shaded blue area represent the optical stimulation period. The brightness of blue shaded area indicates light intensity. **(D1,D2,E)** Dependence of ENG characteristics on light intensity during biased optical stimulation. Average of burst duration difference of L2 **(D1)** and L5 **(D2)** between before and during optical stimulation under low, medium and high stimulation intensity during drug-induced locomotor-like activity (low *n* = 11, medium *n* = 13, high *n* = 9). ^∗^ Indicates 0.01 < *P* < 0.05, ^∗∗^ indicates 0.001 < *P* < 0.01. ^†^ indicates 0.01 < *P* < 0.05, ^††††^ indicates *P* < 0.0001. Linear regression slope is orange for ipsilateral L5 and light green for contralateral L5 and red for ipsilateral L2 and blue for contralateral L2. **(E)** Step cycle period difference between ipsilateral and contralateral L5 under low, medium and high stimulation intensity during drug-induced locomotor-like activity (low *n* = 11, medium *n* = 13, high *n* = 9). ^††††^ indicates *P* < 0.0001, ^∗∗^ indicates 0.001 < *P* < 0.01. Slope of contralateral L5 burst duration and step cycle difference are significantly non-zero.

We also noticed a dose-response relationship between stimulation intensity and the prolongation of the contralateral cycle period and L5 burst duration (Figures 4A–C). At the highest applied light intensity, the rhythm of the contralateral cord can be suppressed, resulting in almost sustained extensor activity (Figure 4C). To further evaluate the relation between the asymmetric changes of the fictive locomotor activity in two sides of the spinal cord and the imbalanced activation intensity of V3 neurons, we systematically manipulated the focal size and light intensity to study the response to three levels of stimulation (low, medium and high intensity; see methods). Interestingly, the L5 burst duration on the contralateral side to the light stimulation showed a positive linear correlation to the optical stimulation intensity (Figures 4D1,D2; *R*^2^ = 0.5081, *P* < 0.0001), but not the ipsilateral L5 (*R*^2^ = 0.007813) or both L2s (*R*^2^ = 0.003418, *R*^2^ = 0.1186 for ipsilateral and contralateral sides, respectively). In turn, the changes of step cycle of contralateral locomotor activities also showed a positive linear correlation with the optical stimulation intensity (Figure 4E; *R*^2^ = 0.4842, *P* < 0.0001). This result indicates that the asymmetric response is dependent on the activation of V3 neurons on one side of the spinal cord.

Together, our experiments showed that the activation of V3 neurons increased extensor activity, and prolonged burst and step cycle duration predominantly on the contralateral side with a smaller effect on the ipsilateral side. These resulted in left-right asymmetric rhythmic activity with lower-frequency bursting on the contralateral than on the ipsilateral side, suggesting that V3 neurons mainly affect the extensor activity of the contralateral CPG.

### Modeling Left–Right Interactions Between Rhythm Generators

To further delineate the function and connectivity of the spinal V3 interneurons involved in left–right coordination, we developed a computational model of the lumbar locomotor circuitry. We built upon our previous model (Shevtsova et al., 2015) with the assumption that V3 neurons slow down the contralateral rhythm by exciting the extensor centers of the contralateral rhythm generators and transsynaptically inhibiting the flexor centers. Our goal was to update the model so that it could reproduce the effect of bilateral and unilateral stimulation of V3 neurons revealed in the above experiments without disrupting its ability to reproduce previous experimental findings concerning the effects of ablation of V0_V_, V0_D_ and all V0 CINs on the left-right coordination (Talpalar et al., 2013).

#### Model Schematic

**FIGURE 5:**
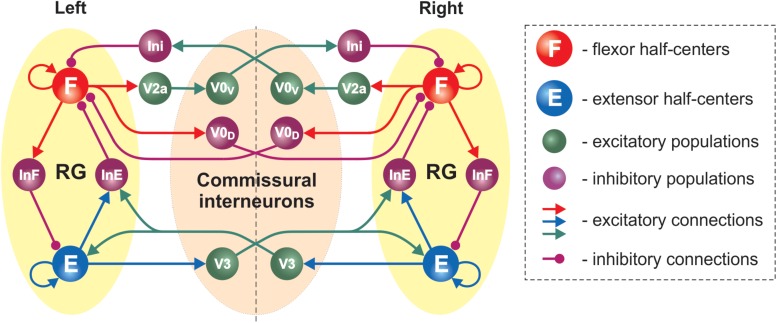
Model schematic of the bilateral spinal circuits consisting of two interconnected rhythm generators. Spheres represent neural populations and lines the synaptic connections between them. The rhythm generator (RG) in each side includes flexor and extensor centers (F and E, respectively) interacting via the inhibitory InF and InE populations. The left and right RGs interact via commissural interneurons (V0_D_, V0_V_, and V3).

The updated model consisted of two rhythm generating networks (RGs), one for each side of the spinal cord (Figure 5). Each RG included two excitatory populations, representing flexor (F) and extensor (E) RG centers that mutually inhibited each other through populations of inhibitory interneurons (InF and InE). Similar to our previous model (Shevtsova et al., 2015), all neurons in both RG centers included a persistent (slowly inactivating) sodium current, allowing them to intrinsically generate rhythmic bursting. Because of the mutual excitation between the neurons within each center, which synchronized their activity, each center could intrinsically generate a population rhythmic bursting activity. However, according to the setup of initial neuronal excitability, under normal conditions, the extensor centers, if uncoupled, expressed sustained activation and the rhythmic activity of each RG were defined by the activity of flexor centers, which then provided rhythmic inhibition of the corresponding extensor centers via the InF populations (see Figure 5 and Zhong et al., 2012; Rybak et al., 2015; Shevtsova et al., 2015; Shevtsova and Rybak, 2016). Interactions between F and E centers of the left and right RGs were mediated by populations of V3, V0_V_ and V0_D_ CINs (Figure 5). Drug-induced fictive locomotion was modeled by an unspecific increase of the excitability of all neurons in the network through a depolarization of the leakage reversal potentials in each neuron. The drug concentration was defined by the parameter α (see section Materials and Methods).

In the present model, the organization of interactions between left and right RGs mediated by V0_V_ and V0_D_ populations of CINs followed that of our previous models (Shevtsova et al., 2015; Danner et al., 2016, 2017; Shevtsova and Rybak, 2016; Ausborn et al., 2019). Specifically (see Figure 5), the populations of inhibitory V0_D_ CINs provided direct mutual inhibition between the left and right flexor centers of the RGs, while the populations of excitatory V0_V_ CINs mediated mutual inhibition between the same flexor centers through oligosynaptic pathways (each V0_V_ population received excitation from the ipsilateral flexor center through a local population of V2a neurons and inhibited the flexor center of the contralateral RG through a population of local inhibitory neurons, Ini). Both V0_V_ and V0_D_ pathways ensured left-right alternation.

The organization of V3 CIN pathways in the present model differed from the previous models and was constructed to fit our experimental results. Based on these results we suggested that V3 populations mediate mutual excitation between the extensor centers of the left and right RGs (Figure 5) and promoted inhibition of the contralateral flexor centers.

Each population of neurons in our model (Figure 5) consisted of 50–200 neurons (Table 1). All neurons were modeled in the Hodgkin-Huxley style (for details see Materials and Methods, section Computational Modeling and Table 1). Heterogeneity within the populations was ensured by randomizing the baseline value for leakage reversal potential and initial conditions for the values of membrane potential and channel kinetics variables. Connections between the populations were modeled as sparse random synaptic connections. Model equations and simulation procedures are listed in Section “Materials and Methods.” Population specific parameters are listed in Table 1 and connection weights and probabilities are specified in Table 2.

**TABLE 1:** Number of neurons and neuron parameters in different populations.

**Neuron type**	**N, number of neurons**	**${\bar{g}}_{N\,a}$, mS/cm^2^**	**${\bar{g}}_{N\,a\,P}$, mS/cm^2^**	**${\bar{g}}_{K}$, mS/cm^2^**	***g*_*L*_, mS/cm^2^**	**${\bar{E}}_{L\,O}$, mV**
F	200	25	0.75(±0.00375)	2	0.07	−67(±0.67)
E	100	25	0.75(±0.00375)	2	0.07	−60(±0.6)
InF	100	10		5	0.1	−67(±1.34)
InE	100	10		5	0.1	−72(±1.44)
V0D	50	10		5	0.1	−68(±2.04)
V2a	50	40		5	0.8	−60.5(±1.21)
V0V	50	10		5	0.1	−62(±1.24)
Ini	50	10		5	0.1	−64(±1.28)
V3	100	10		5	0.1	−68(±2.04)

**TABLE 2:** Average weights ${\bar{w}}_{j\,i}$ and probabilities (*p*) of synaptic connections.

**Source**	**Target populations**
**population**	**(${\bar{w}}_{j\,i}$, probability of connection *p*)**
i-F	i-F(0.009, *p* = 0.1);
	i-InF(0.01, *p* = 0.1);
	i-V2a(0.01, *p* = 0.1);
	i-V0_D_(0.06, *p* = 0.1)
i-E	i-E(0.018, *p* = 0.1);
	i-InE(0.12, *p* = 0.1);
	i-V3(0.2, *p* = 0.05)
i-InF	i-E(−0.15, *p* = 0.1)
i-InE	i-F(−0.3, *p* = 0.1)
i-Ini	i-F(−0.5, *p* = 0.1)
i-V2a	i-V0_V_(1.5, *p* = 0.1)
i-V0_V_	c-Ini(1.5, *p* = 0.1)
i-V0_D_	c-F (−0.18, *p* = 0.1)
i-V3	c-E(0.05, *p* = 0.05);
	c-inE(1, *p* = 0.05)

*Prefixes i- and c- indicate ipsi- and contralateral populations.*

#### The Model Exhibits Characteristic Features of Drug-Induced Fictive Locomotion and Frequency-Dependent Changes of Left–Right Coordination Following Removal of V0 Commissural Interneurons

**FIGURE 6:**
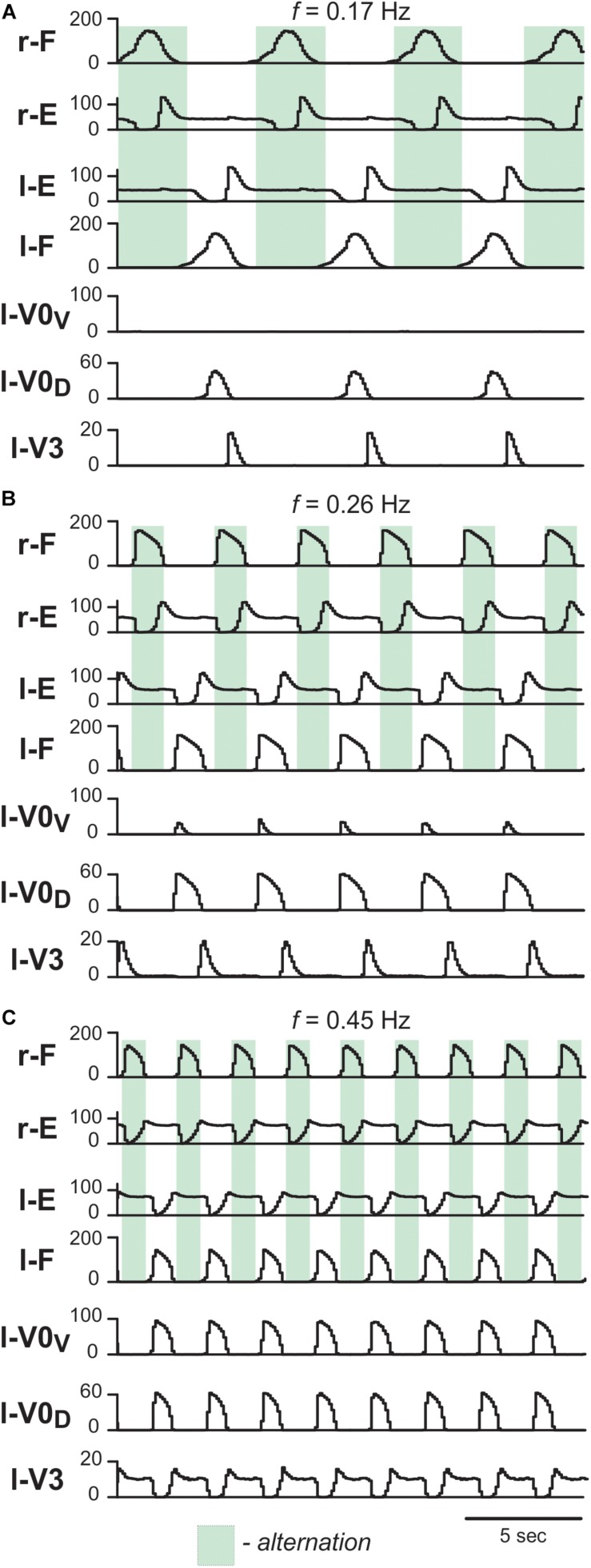
Model performance under normal conditions. Integrated population activities of flexor (F) and extensor (E) rhythm generator centers and left commissural interneurons are shown at low (**A**; α = 0.01), medium (**B**; α = 0.03), and high excitation levels (**C**; α = 0.09). Activities of populations in this and following figures are shown as average histograms of neuron activity [spikes/(N × s), where N is a number of neurons in population; bin = 100 ms]. Shaded green areas show intervals of right flexor activities. r-: right; l-: left.

First, we characterized the model performance under normal conditions by simulating drug-induced fictive locomotion (Figure 6). The model exhibited alternation between the rhythmic activities of the flexor and extensor RG centers on each side as well as alternation between the activities of the left and right RGs. By increasing α (simulating an increase in the drug concentration) the burst frequency increased (Figures 6A–C). The frequency increase was accompanied by an asymmetric decrease of the burst durations: extensor burst durations decreased more than flexor burst durations. At all frequencies, left–right alternation was maintained. Thus, the model reproduced the main characteristics of drug-induced fictive locomotion in mice.

**FIGURE 7:**
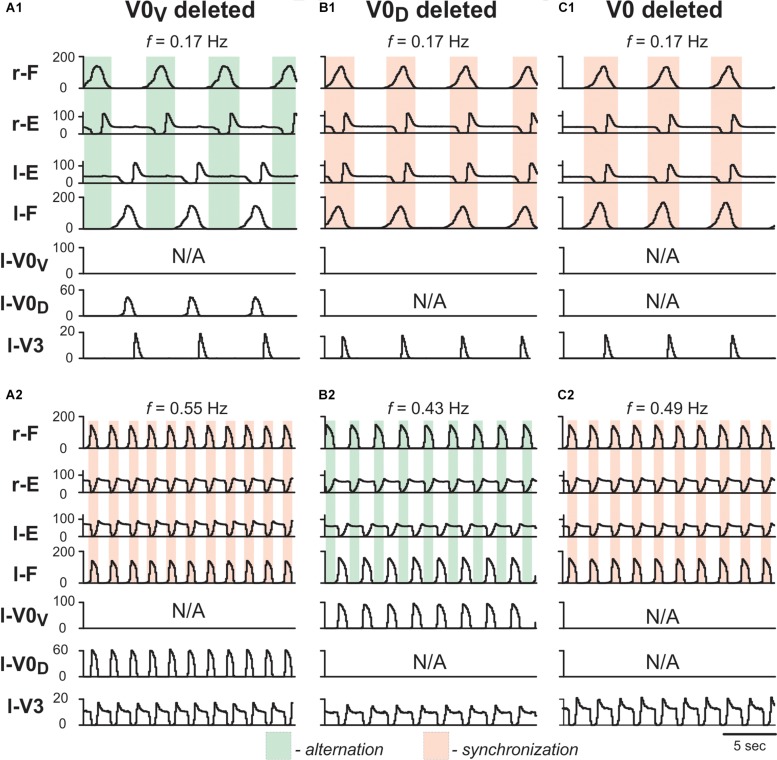
Model performance after removal of V0_V_, V0_D_ and all V0 commissural interneurons. Integrated population activities of flexor and extensor rhythm generator centers (F and E, respectively) and left commissural interneurons are shown after removal of V0_V_ **(A1,A2)**, V0_D_ **(B1,B2)**, and all V0 **(C1,C2)**, at a low (**A1,B1,C1**; α = 0.01) and a high (**A2,B2,C2**; α = 0.08) excitation level and oscillation frequency. Shaded areas show intervals of right flexor activities. Green indicates left–right alternation and pink left–right synchronization. r-: right; l-: left.

To test whether the model is still consistent with the frequency-dependent changes in left–right coordination following the removal of V0 CINs (Talpalar et al., 2013), we simulated the selective removal of V0_V_, V0_D_ or both V0 CIN populations by setting all connection weights from the selected types of neurons to 0. Removal of V0_V_ CIN populations did not change left-right alternation at low locomotor frequencies (Figure 7A1), but demonstrated left-right synchronized activity at high oscillation frequencies (Figure 7A2). Removal of V0_D_ CINs had the opposite effect: left-right synchronization occurred at low frequencies (Figure 7B1), while left-right alternation was maintained at high frequencies (Figure 7B2). Finally, removal of both types of V0 CIN populations led to left-right synchronization at all frequencies (Figures 7C1,C2). Thus, similar to the previous model (Shevtsova et al., 2015) the present model was able to reproduce the experimental results on the speed-dependent role of V0_V_ and V0_D_ in support of left-right alternation (Talpalar et al., 2013).

#### The Model Reproduces Deceleration of the Rhythm by Tonic Stimulation of V3 Neurons

**FIGURE 8:**
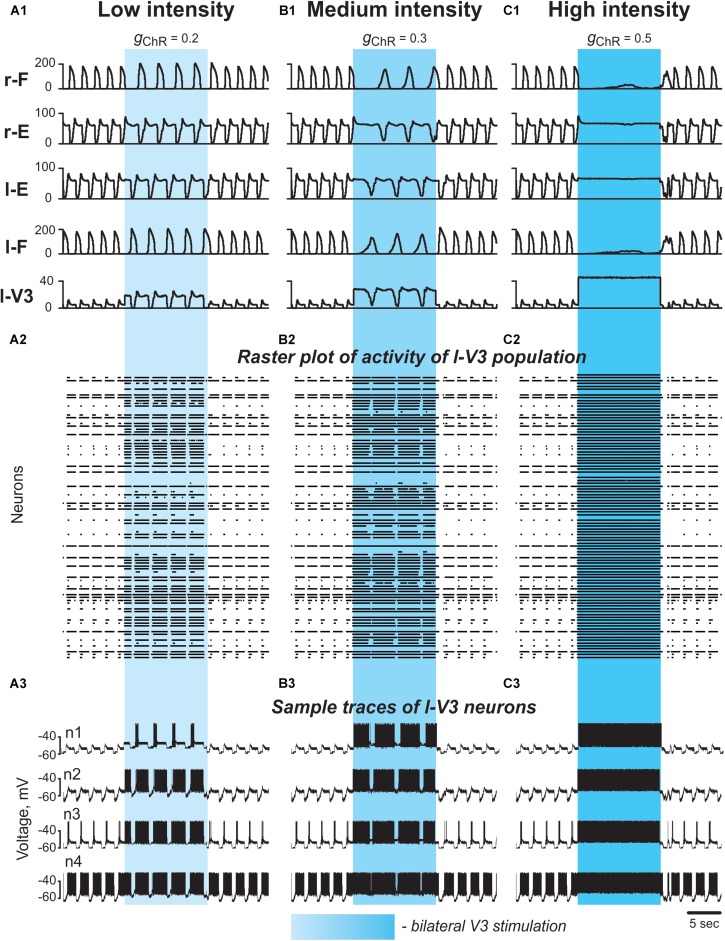
Effect of bilateral stimulation of V3 commissural interneurons during fictive locomotion in the model. **(A1–A3)** Low V3-stimulation intensity (*g*_*ChR*_ = 0.2). **(B1–B3)** Medium V3-stimulation intensity (*g*_*ChR*_ = 0.3). **(C1–C3)** High V3-stimulation intensity (*g*_*ChR*_ = 0.5). Stimulation was applied to all V3 neurons (on both sides of the cord). For all stimulations fictive locomotion was evoked at α = 0.06. **(A1,B1,C1)** Integrated population activities of the flexor and extensor rhythm generator centers and the left V3 population. **(A2,B2,C2)** Raster plots of spikes elicited by neurons in the left V3 population. **(A3,B3,C3)** Traces of the membrane potential of sample neurons in the left V3 population with different excitability. Shaded blue areas show the interval when V3 stimulation was applied. r-: right; l-: left.

**FIGURE 9:**
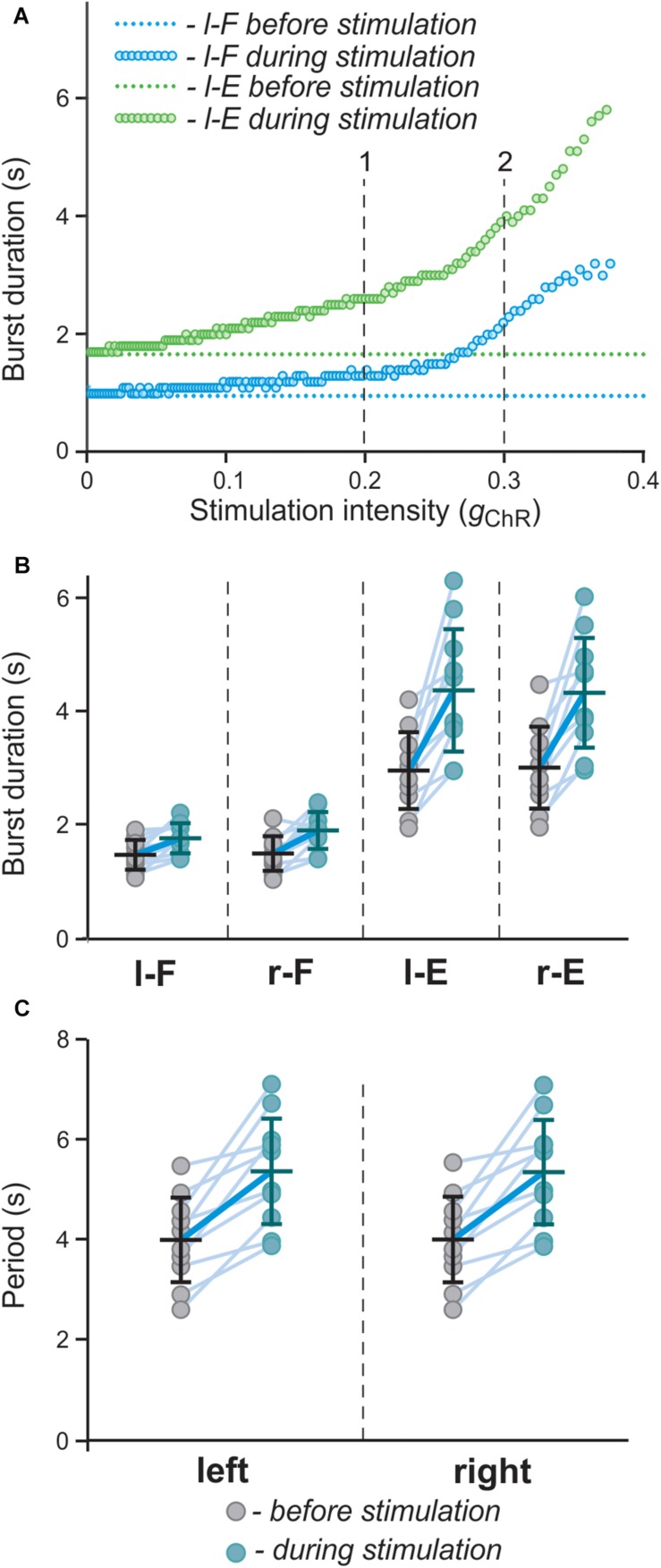
Effect of bilateral stimulation of V3 interneurons on flexor and extensor burst duration s and oscillation period in the model. **(A)** Dependence of flexor and extensor burst duration on stimulation intensity (*g*_*ChR*_; shown for the left side only). Intensity of stimulation was gradually changed from 0 to 0.4. The vertical lines (1 and 2) correspond to simulations shown in Figures 8A1,A2, respectively. **(B,C)** Flexor and extensor burst duration and period of oscillations in a series of model experiments (*n* = 12) with randomly chosen parameters α and *g*_*ChR*_ before (gray circles) and during (blue circles) bilateral stimulation of V3 populations. Each circle represents the average value of estimated parameter for one simulation. Light blue lines connecting two circles represent parameter increase in one simulation Thick, dark blue line represent the average of increase.

To simulate bilateral optogenetic stimulation of V3 CINs (see Section Optical Activation of Lumbar V3 Interneurons Increases the Intensity of Extensor Motor Activity and Slows Oscillation Frequency of Drug-Evoked Fictive Locomotion), we incorporated a channelrhodopsin ionic current, *I*_*ChR*_, in V3 neurons (see Materials and Methods, Section Simulations of Changes in the Locomotor Frequency by Neuroactive Drugs and Application of Photostimulation), which was activated in all V3 neurons for 15 s during ongoing locomotor activity. The results of these simulations with progressively increased stimulation intensity are shown in Figures 8, 9. At any value, the applied stimulation increased the firing rate of active V3 neurons and recruited new neurons that were silent before stimulation (Figures 8A2–C2,A3–C3). Immediately with its onset, V3-stimulation increased the cycle period and reduced the burst frequency of both RGs (Figures 8A1,B1). The frequency reduction was mainly caused by a prolongation of the extensor burst duration. Flexor-extensor and left–right alternation were preserved during this stimulation. With increasing value of stimulation, the frequency was progressively decreased (Figures 8A1,B1). At high stimulation values, the rhythm could be stopped, resulting in sustained activation of both extensor centers and suppression of both flexor centers (Figure 8C1). Once the stimulation was stopped, the model exhibited a short transitional period (one or two cycles) and then returned to the same burst frequency and pattern that were expressed before stimulation.

To study the effect of bilateral stimulation intensity on model behavior in more detail, we performed a simulation when the value of *g*_*ChR*_ (conductance of the channelrhodopsin ionic current, *I*_*ChR*_, that characterizes the intensity of photostimulation in the model) was slowly changed from 0 to 0.4 (see section Materials and Methods). The results of this simulation (Figure 9A) show that both flexor and extensor phases increase with increasing intensity of stimulation and the slope of this increase is higher for larger values of *g*_*ChR*_. To simulate experimental variability, in a series of simulations parameters α (defining the average level of neuron excitation in the model) and *g*_*ChR*_ were randomly chosen from a uniform distribution in intervals [0.01; 0.06] and [0.18; 0.4], respectively. For each pair (α, *g*_*ChR*_) a simulation was run in which average flexor and extensor burst durations and period of oscillations were calculated and compared to the control condition (*g*_*ChR*_ = 0; see Figures 9B,C). These results qualitatively reproduce the experimental results shown in Figures 2D,E.

Altogether our results show that the model closely reproduces our experimental findings of bilateral optogenetic stimulation of V3 neurons during drug-induced fictive locomotion when stimulation was applied at the midline and equally affected left and right V3 neurons (see section Optical Activation of Lumbar V3 Interneurons Increases the Intensity of Extensor Motor Activity and Slows Oscillation Frequency of Drug-Evoked Fictive Locomotion).

The reduction of the oscillation frequency when V3 neurons were stimulated occurred because both (left and right) extensor centers were activated by V3 neurons and they both provided an additional inhibition to the corresponding flexor centers through the corresponding inhibitory populations (InE), which reduced the average excitation of the flexor centers (Figure 5). In addition, activated V3 neurons provided direct excitation of the contralateral InE populations inhibiting the corresponding flexor centers. Note that the frequency of persistent sodium current-dependent oscillations positively correlates with the average excitation of a population of neurons with this current and mutually excitatory interconnection (Butera et al., 1999; Rybak et al., 2004, 2015). Since the rhythm in our model was generated by flexor centers, the reduction of their excitation during V3 neuron activation led to the reduction of oscillation frequency generated in both RGs. Furthermore, the reciprocal excitation of the extensor centers through V3 CINs created a positive feedback loop that amplified the firing rates of its constituent neurons and consequently the net inhibition exerted on the flexor centers.

#### The Model Reproduces Asymmetric Changes of the Locomotor Rhythm by Unilateral Stimulation of V3 Neurons

**FIGURE 10:**
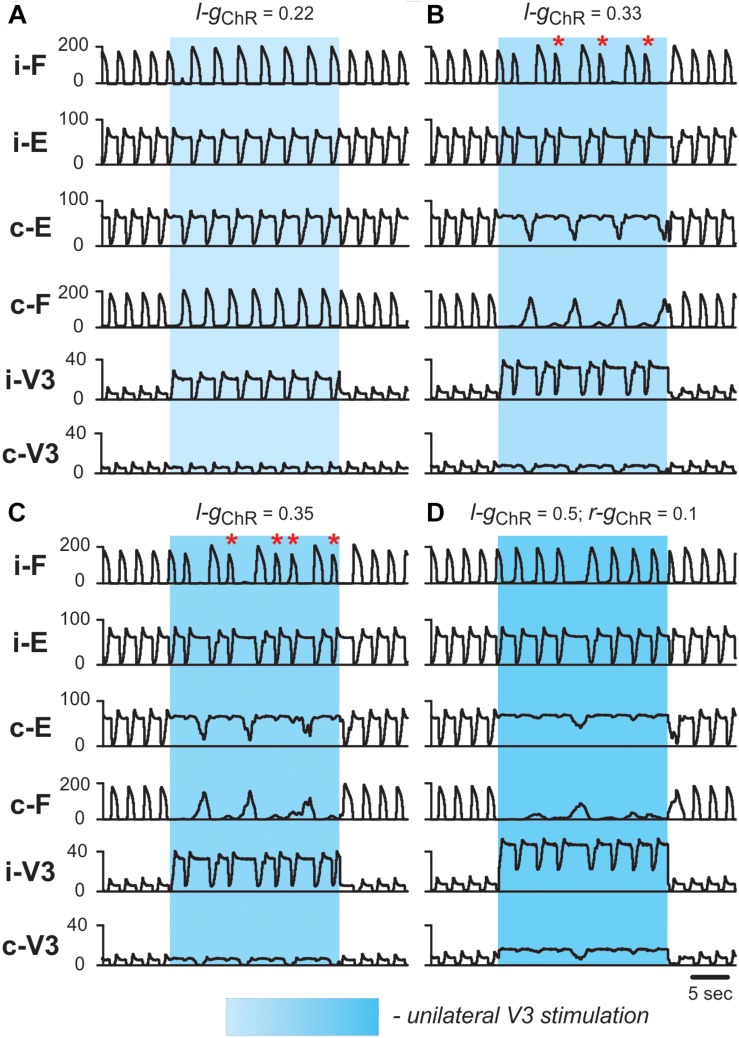
Effect of unilateral stimulation of V3 commissural interneurons during fictive locomotion in the model. Fictive locomotion was evoked at α = 0.065. All panels show integrated population activities of the flexor (F) and extensor (E) rhythm generator centers and the V3 populations ipsi- and contralateral to the stimulation. Shaded blue areas show the interval when V3-stimulation was applied. Four different stimulation intensities are shown: **(A)** *g*_*ChR*_ = 0.22; **(B)** *g*_*ChR*_ = 0.33; **(C)** *g*_*ChR*_ = 0.35; **(D)** *g*_*ChR*_ = 0.5 in ipsilateral and *g*_*ChR*_ = 0.1 in contralateral V3 population. Red asterisks mark additional flexor bursts on ipsilateral side. i-: ipsilateral; c-: contralateral.

To simulate the effects of unilateral activation of V3 neurons during locomotor activity (see section Biased Optical Activation of V3 Interneurons in Spinal Segment L2 Leads to Asymmetrical Left–Right Motor Activity), we activated *I*_*ChR*_ in all V3 neurons located on one side of the cord. At a low value of unilateral activation of ipsilateral V3 population (Figure 10A), the extensor burst durations and the cycle periods increased on both sides, while the flexor-extensor and left-right alternation remained unchanged, which was similar to the effect of bilateral stimulation. With increasing value of stimulation (Figures 10B,C), the rhythm of the contralateral circuits was progressively slowed down, and additional bursts appeared ipsilateral to the stimulation. Figure 10B shows a stable 2-to-1 relationship between the number of burst ipsilateral and contralateral to the stimulation. In Figure 10C one can see both 2-to-1 and 3-to-1 relationships. In all cases, reciprocity between the flexor centers maintained. Our simulations shown in Figures 10B,C qualitatively reproduced the experimental results concerning the response of the fictive locomotor pattern to the weak and medium unilateral optical stimulation of V3 neurons (Figures 4A,B) where one can also see an unequal number of flexor bursts on ipsi- and contralateral sides (marked by asterisks in Figures 10B,C).

**FIGURE 11:**
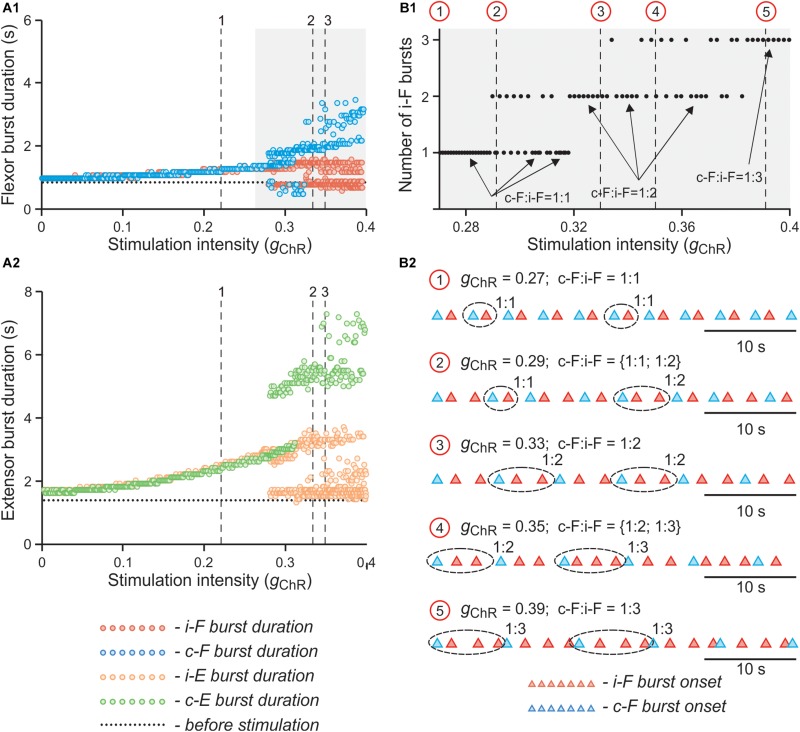
Effect of unilateral stimulation intensity of V3 interneurons on flexor and extensor burst durations and oscillation period in the model. **(A1,A2)** Dependence of flexor **(A1)** and extensor **(A2)** burst durations on intensity of stimulation (*g*_*ChR*_) when stimulation was applied to the left V3 population. Intensity of stimulation was gradually changed from 0 to 0.4. The vertical lines (1, 2 and 3) correspond to simulations shown in Figures 10A–C, respectively. **(B1,B2)** Dependence of number of additional ipsilateral flexor bursts during single contralateral period on parameter *g*_*ChR*_ at higher values of stimulation intensity In **(B1)**, *g*_*ChR*_ was gradually changed from 0.27 to 0.4. Arrows indicate 1:1, 1:2, and 1:3 ratios between the number of ipsi- and contralateral flexor bursts. Red circles and vertical lines indicate selected values of *g*_*ChR*_ for which additional simulations were run when *g*_*ChR*_ was fixed. In **(B2)**, the timing of flexor burst onsets for these simulations are shown. The corresponding values of *g*_*ChR*_ for each simulation are indicated above each trace. Traces are shown for 40 s of simulated time. i-: ipsilateral; c-: contralateral; F - flexor rhythm generator center.

This effect is even more obvious on the bifurcation diagrams in Figures 11A1,A2 that show flexor and extensor phase durations plotted against parameter *g*_*ChR*_, which has been gradually increased from 0 to 0.4. At lower values of *g*_*ChR*_, both flexor and extensor phases progressively increase with an increase of parameter *g*_*ChR*_. At a critical value of *g*_*ChR*_, the curves characterizing dependence of flexor and extensor phase durations on *g*_*ChR*_ bifurcate into two and then three branches. The bifurcation diagram in Figure 11B1 demonstrates that the stable regime at lower *g*_*ChR*_ values is characterized by a 1:1 ratio between the numbers of bursts in the contra- and ipsilateral flexor centers and then with increasing *g*_*ChR*_ the model starts to exhibit also 1:2 and 1:3 burst ratios with multiple bursts of the ipsilateral flexor center during one period of contralateral oscillations. In the diagrams in Figures 11A1,B1 the bifurcations (transitions between regimes) can be seen as discontinuities. Transitions between stable regimes of 1:1, 1:2, and 1:3 ratios happen through intermediate quasiperiodic regimes where 1:1 and 1:2 or 1:2 and 1:3 ratios between the numbers of contra- and ipsilateral bursts occur and alternate with different integer intervals. To assess transition between regimes in more detail, in a series of simulations we fixed parameter *g*_*ChR*_ at some values (marked by red circles and vertical dashed lines in Figure 11B1) and estimated the timing of burst onsets in ipsi- and contralateral flexor centers for 40 s of simulated time. The results of these simulations are shown as the diagrams of ipsi- and contralateral flexor burst onsets in Figure 11B2. These diagrams demonstrate how the 1:1 regime at lower values of *g*_*ChR*_ transitions to the 1:2 regime through an intermediate quasiperiodic {1:1; 1:2} regime as *g*_*ChR*_ increases, and then to the 1:3 regime though another quasiperiodic {1:2; 1:3} regime.

**FIGURE 12:**
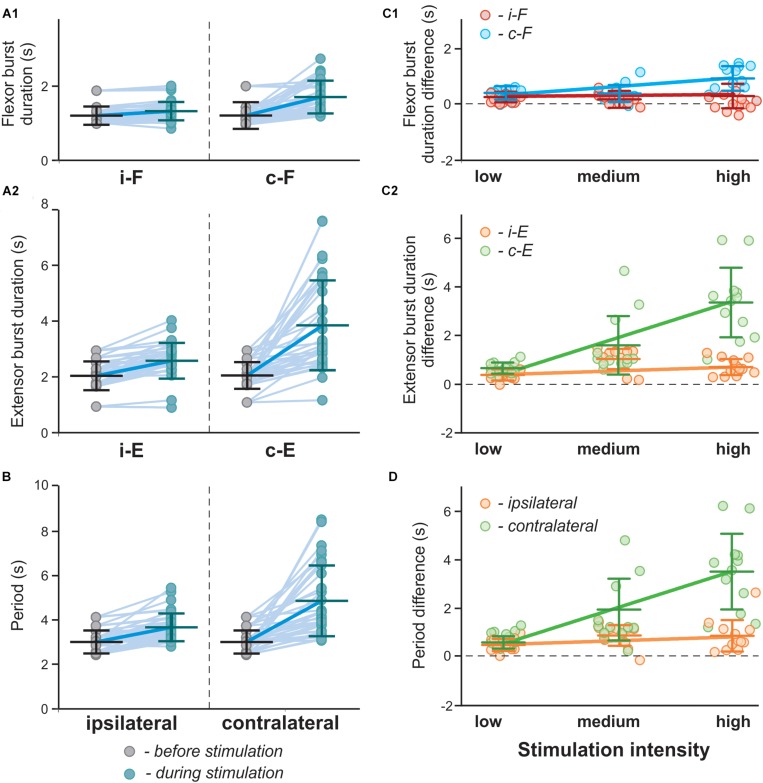
Effect of unilateral stimulation of V3 interneurons on flexor and extensor burst durations and oscillation period in the model. **(A1,A2,B)** Flexor **(A1)** and extensor **(A2)** burst duration and period of oscillations **(B)** in a series of model experiments with randomly chosen parameters α and *g*_*ChR*_ before (gray circles) and during (blue circles) stimulation of the left V3 populations. Each circle represents the average value of estimated parameter for one simulation. Light blue lines connecting two circles represent parameter change in one simulation Thick, dark blue line represent the average of change. **(C1,C2,D)** Differences in flexor **(C1)** and extensor **(C2)** burst duration and period of oscillations **(D)** for the above modeling experiments estimated according to stimulation intensity. Low stimulation intensity corresponds to *g*_*ChR*_ ∈ [0.18,0.25], medium, *g*_*ChR*_ ∈ [0.26,0.33], in stimulations with high intensity *g*_*ChR*_ ∈ [0.33,0.4]. Linear regression slopes are orange for ipsilateral and green for contralateral extensor centers and red for ipsilateral and blue for contralateral flexor extensor centers. i-: ipsilateral; c-: contralateral.

The 1:2, 1:3 and intermediate quasiperiodic regimes are characterized by increased variability of burst durations during a single recording (see Figures 11A1,A2). Such behavior obviously affects the estimated average phase duration and period in particular experiments. Indeed, in experimental results shown in Figures 3D1, 4D1, in individual recordings the flexor burst duration decreases during photostimulation as compared to the control condition, while on average there is either an increase (Figure 3D1) or no change (Figure 4D1). This is reproduced in our simulations (Figures 12A1,B), in which the results are shown for a series of computer experiments when parameters α and *g*_*ChR*_ were randomly chosen in intervals [0.01; 0.06] and [0.18; 0.4], respectively, and for each pair (α, *g*_*ChR*_) a single simulation was performed to estimate average values for burst duration and oscillation period and compare with the control condition. These simulation results closely reproduce the experimental results shown in Figures 3D1,E. Interestingly, when the results of these simulations were conditionally separated according to the value of parameter *g*_*ChR*_ and the differences in flexor and extensor burst durations and period of oscillation in stimulated vs. control conditions were plotted against stimulation strength (similar to what was done in experiment in the spinal cord *in vitro*, see Figures 4D1,E), these results strikingly resembled the experimental results in Figures 4D1,E and demonstrated asymmetric response in the ipsi- and contralateral RG centers. Altogether, our modeling results strongly support the hypothesis that activation of V3 interneurons on one side of the spinal cord more strongly impacts the contralateral circuits.

To simulate the effects of high intensity and larger focal area unilateral stimulation, an additional weak stimulation of the contralateral V3 neuron population was applied (Figure 10D). In this case, the rhythmic activity on the ipsilateral side slowed down during the stimulation and the contralateral extensor centers became constantly active, while irregular low-amplitude activity of the contralateral flexor centers occurred. This behavior of the model was similar to the results of experimental studies at a high intensity unilateral stimulation when the rhythm of the contralateral cord was suppressed, resulting in almost sustained extensor activity (Figure 4C). Based on our simulation results, we suggest that at high stimulation intensities the stimulation partly affected V3 neurons on the side contralateral to stimulation.

## Discussion

In the current study, we combined experimental studies with computational modeling to investigate the potential roles that V3 interneurons play in the spinal locomotor network. In our experiments, we used the *in vitro* preparations of isolated spinal cords from neonatal (P2-P3) mice in which fictive locomotion was induced by a mixture of neuroactive drugs (NMDA and 5-HT). Although the isolated neonatal spinal cords are characterized by a lack of supra-spinal and sensory inputs and are from young animals whose weight bearing ability is not fully developed, this preparation allows studying basic CPG circuits and functional interactions between genetically identified spinal interneurons (Kjaerulff and Kiehn, 1997; Lanuza et al., 2004; Gosgnach et al., 2006; Zhang et al., 2008). Using optogenetic approaches, we were able to selectively activate V3 interneurons at different regions of the spinal cord during drug-evoked fictive locomotion. Our computational model of locomotor circuits was able to qualitatively reproduce the experimental results. Our study revealed that lumbar V3 interneurons strongly enhance the contralateral extensor activity and regulate the frequency of the locomotor oscillations. We suggest that spinal V3 CINs mediate mutual excitation between the extensor centers of the left and right rhythm generators in the lumbar spinal cord, which might support the synchronization of left-right activity under certain conditions during locomotion. In addition, we show that unilateral activation of V3 CINs can produce left-right asymmetrical rhythmic outputs, which provides a potential mechanism for the separate regulation of left–right limb movements as required for changing direction or during split-belt locomotion (Kiehn, 2016).

### Optogenetic Activation of V3 Interneurons

Mapping the functional connectivity among neurons in the central nervous system is vital to understand circuit mechanisms underlying behavior. This is especially true within the spinal cord, which generates the rhythmic and patterned motor outputs necessary for coordinated movement (Grillner, 2006; Kiehn, 2006, 2011; Danner et al., 2015). However, uncovering the functional circuitry within the spinal cord has proven difficult due to a lack of clear anatomical organization compared to other brain regions. In the current study, novel optogenetic tools (Dougherty et al., 2013; Hägglund et al., 2013; Levine et al., 2014) have enabled us to specifically, reversibly, and acutely regulate the activity of a molecularly identified group of spinal neurons. By using Sim1cre-Ai32 mice (Chopek et al., 2018), we were able to target V3 interneurons on both or just one side of the isolated spinal cord, allowing us to start dissecting the function of V3 CINs in spinal locomotor circuits.

Using whole-cell patch-clamp recordings, we could verify that all ChR2-EYFP-expressing cells in the lumbar spinal slices could be exclusively depolarized by blue fluorescent light and elicit spikes during periods of the light pulses (10–20 s). During our functional study, however, we had to keep the whole lumbar cord intact and deliver the LED light on the ventral surface of the isolated spinal cord. Thus, we expect to have activated V3 neurons that had cell bodies and terminals located or neurites passing the illuminated region that is relatively close to the ventral surface. Although we were not able to specify the particular group of V3s, which we might be activating, we kept the light intensity, size and position consistent to limit variability across experiments.

### The V3 CINs Provide Excitation to the Contralateral CPG Extensor Center

The identification of genetically defined interneuron populations has enabled us to characterize their specific functions in locomotor behaviors (Goulding, 2009; Gosgnach et al., 2017; Deska-Gauthier and Zhang, 2019). Until now, however, most experimental data were obtained by genetically eliminating certain classes of spinal interneurons without clear understanding of connectivity among the spinal interneurons (Lanuza et al., 2004; Gosgnach et al., 2006; Crone et al., 2008; Zhang et al., 2008; Talpalar et al., 2013; Bellardita and Kiehn, 2015; Haque and Gosgnach, 2019). In the case of V3 interneurons, genetic deletion of entire V3 population reduced the general robustness of the rhythmicity of the *in vivo* and *in vitro* locomotor activity and led to unstable gaits (Zhang et al., 2008). Our previous anatomical studies were only able to show that V3 cells had broad projections to contralateral motor neurons and other ventral spinal interneurons along the lumbar spinal cord, but we still do not know their precise anatomical position and their role in the CPG network (Zhang et al., 2008; Blacklaws et al., 2015). The current spatially controlled activation of V3 neurons was the first step to overcome this limitation to decipher their functional role in the spinal cord.

We found that the bilateral photoactivation of V3 neurons in the isolated spinal cord during fictive locomotion increased the intensity and duration of L5 bursts and prolonged the step cycles in all recorded lumbar roots whereas the duration of bursts in L2 roots was not significantly affected. These results suggested that, under our experimental condition, the photoactivated V3 interneurons predominantly excited the circuits responsible for extensor activities, which in turn reduced the frequency of fictive locomotion oscillations. This conclusion became more evident when we activated V3 interneurons only on one-side of the cord with varied light intensities. With such stimulations, we found that only the increase of burst duration of contralateral L5 were positively correlated to the intensity of the illuminating light, and at high intensity of unilateral photostimulation the contralateral L5 activity could express a sustained activity. More interestingly, such uneven regulation of contralateral extensor excitability by V3 CINs significantly decreased the bursting frequency of the contralateral ventral root output, which led to asymmetrical oscillation of the motor outputs on the two sides of the spinal cord. Thus, our experimental results support the conclusion that the lumbar V3 interneurons, or at least a sub-group of them, strongly regulate the extensor activity and step cycle duration on the contralateral side of the cord.

Similar to other genetically defined types of spinal interneuron populations (Bikoff et al., 2016; Gosgnach et al., 2017; Deska-Gauthier and Zhang, 2019), the V3 population is heterogeneous and consists of sub-populations with different anatomical, physiological and molecular profiles (Borowska et al., 2013, 2015). In the present study, however, we did not consider different subtype of V3 neurons. When we delivered the LED fluorescent light on the ventral surface of the spinal cord, we most likely activated a mixed group of different V3 neuron types in the illuminated region. Nonetheless, this discovery has provided direct evidence that some subtypes of V3 neurons can regulate the rhythm and pattern of the locomotor output through manipulating the contralateral extensor circuits.

### Model Prediction: V3 Interneurons Project to Local Contralateral Inhibitory V1 Neurons

To reproduce the experimental results, it was necessary to model connections from the V3 CINs to the local contralateral inhibitory populations that inhibit the contralateral flexor centers (InE; Figure 5). The main function of these connections in the present model was to allow for the reduction in frequency with V3 stimulation. In our previous models (Danner et al., 2016, 2017), we had introduced hypothetical inhibitory commissural interneuron populations mediating inhibition from the extensor centers to the contralateral flexor centers. These connections allowed for perfect alternation (0.5 normalized phase difference) between the left and right RGs. In the present model, the V3 connection to the local contralateral inhibitory neurons achieved the same function while no additional populations were needed.

The classes of V1 and V2b inhibitory neurons have been implicated in mediating flexor-extensor alternation (Zhang et al., 2014). Specifically, V1 neurons were suggested to provide inhibition from the extensor to the flexor RGs (Zhang et al., 2014; Shevtsova and Rybak, 2016): the same connection pattern as the InE populations in our model (Figure 5). Thus, our model predicts that V3 CINs project to contralateral V1 neurons that inhibit the flexor center. This prediction awaits experimental verification.

### Coordination of Left–Right Activities by V3 Interneurons During Locomotion

Until now, several types of CINs, including V0, V3, and dI6, have been identified in the ventral spinal cord (Lanuza et al., 2004; Zhang et al., 2008; Andersson et al., 2012; Talpalar et al., 2013; Bellardita and Kiehn, 2015; Haque et al., 2018). When each of these CIN types, except V3, was genetically deleted, the left–right hind-limb alternation was affected. Particularly, V0_V_ and V0_D_ CINs were shown to secure the speed-dependent left-right alternation at walking and trotting gaits without effects on hind-limb synchronization observed during gallop and bound (Talpalar et al., 2013; Bellardita and Kiehn, 2015). On the other hand, deletion of V3 CINs did not affect the left-right alternation of the limb movement during walk and trot (Zhang et al., 2008). We had suggested that V3 represent the CIN populations that mediate left–right synchronization in certain conditions (e.g., when some or all V0 CINs are genetically removed) and during gallop and bound. In our previous computational models (Rybak et al., 2013; Shevtsova et al., 2015; Danner et al., 2016, 2017; Shevtsova and Rybak, 2016), to perform this function the V3 CINs were assigned to explicitly mediate mutual excitation between the flexor centers of the left and right rhythm generators. However, our current experimental outcome contradicts this assumption, suggesting functional connections of V3 CINs to the contralateral extensor centers instead of the flexor centers. To match our experimental findings, we changed the connection of simulated V3 populations from the activation of contralateral flexor centers to the activation of contralateral extensor centers. With this change in the V3 connectivity we were able to qualitatively reproduce all our current and previous experimental results, including speed-dependent left–right alternation mediated by V0_V_ and V0_D_.

This unification of the experimental and computational data confirmed that the proposed organization of our current computational model of spinal circuits is plausible, and that V3 interneurons can mediate the synchronization of left-right hind-limb activities through mutual excitation of extensor centers of the left and right spinal rhythm generators.

Excitatory commissural neurons (such as the V3 CINs) have been reported to mediate reticulospinal and vestibulospinal input as well as input from ipsilateral somatosensory afferents to contralateral motoneurons (Bruggencate et al., 1969; Bruggencate and Lundberg, 1974; Matsuyama et al., 2004; Cabaj et al., 2006). Kasumacic et al. (2015) further described a population of commissural interneurons that directly receives vestibulospinal input and contributes to contralateral extensor activity. These pathways are thought to be involved in stabilizing posture during locomotion. Based on our results, the V3 class is a likely candidate for mediating and integrating these inputs. Thus, studying interaction and convergence of supraspinal and somatosensory afferent inputs on V3 might lead to a better understanding of the role and function of these pathways during locomotion.

Activation of V3s of one side of the spinal cord resulted in asymmetric modulation of extensor and flexor phase durations between the two sides of the cord. This allows a suggestion that V3 interneurons may play important functional roles in left–right coordination when changing the direction of movement, walking on a curved path (Courtine and Schieppati, 2003a, b), on unequal surfaces or a split-belt treadmill (Forssberg et al., 1980; Yang et al., 2005; Frigon, 2017). Such locomotor behaviors may be controlled by an additional supraspinal or afferent activation/suppression to V3 neuron populations on one side of the cord. In these situations, different speeds for left-right limbs are necessary for maintaining stable locomotion. The precise mechanisms underlying such movements are still unknown, but it has been indicated that the independent but closely coordinated CPG circuits in both sides of the spinal cord are involved (Frigon, 2017). In our current experimental and modeling studies, we could induce asymmetric rhythmic activities with an unequal number of bursts between the two sides of the spinal cord by unilaterally activating V3 neurons. These results closely resemble the findings in split-belt locomotion with a large speed difference for the left and right belts, in which additional steps were observed on the fast side (Forssberg et al., 1980; Yang et al., 2005; Frigon, 2017). Such activity patterns are reminiscent of those of (weakly-)coupled oscillators, with asymmetric drive (Pikovsky et al., 2001; Pikovsky and Rosenblum, 2003; Rubin et al., 2011) and since our data were obtained in an isolated spinal cord preparation, they provide even more convincing evidence of the existence of a CPG on either side of the cord.

In conclusion, our study provides strong evidence that spinal V3 CINs are involved in left-right limb coordination during locomotion presumably by providing mutual excitation between the extensor centers of the left and right rhythm generators and mediating inhibition from the extensor centers to their contralateral flexor centers through an additional inhibitor interneuron population. In addition, we have shown that unilateral activation of V3 CINs can produce left-right asymmetric rhythmic outputs. Which led us to suggest that V3 neurons are involved in changing the direction of movements and/or in control of locomotion through complex environments.

## Materials and Methods

### Animals

The generation and genotyping of Sim1^Cre/+^ mice were described previously by Zhang et al. (2008). Ai32 mice were from the Jackson Laboratory (Stock No. 012569). It contains Rosa26-cytomegalovirus early enhancer element/chicken beta-actin promoter (CAG)-loxP–Stop codons–3x SV40 polyA–loxP (LSL)-channelrhodopsin2 (ChR2)-enhanced yellow fluorescent protein (EYFP)-woodchick hepatitis virus posttranscriptional enhancer (WPRE; RC-ChR2). Sim1cre-Ai32 mice were generated by breeding these two strains expressed ChR2/EYFP fusion-protein in Sim1 expressing cells. TdTomato Ai14 (Jackson Laboratory Stock No. 007908) conditional reporter (referred as tdTom) mice were generated and genotyped as previously described (Blacklaws et al., 2015). Sim1^Cre/+^; tdTom; Ai32 mice were generated by crossing Sim1^Cre/+^; tdTom mice with Ai32 mice. All procedures were performed in accordance with the Canadian Council on Animal Care and approved by the University Committee on Laboratory Animals at Dalhousie University.

### Electrophysiology and Immunohistochemistry

#### Preparation

All experiments were performed using spinal cords from Sim1cre-Ai32 mice (isolated spinal cord recording and whole-cell patch-clamp recording) and Sim1^Cre/+^; tdTom; Ai32 mice (whole-cell patch-clamp recording) at postnatal day (P) P2-P3. The mice were anesthetized, and the spinal cords caudal to thoracic (T) 8 segments were dissected in an ice-cold oxygenated Ringer’s solution (111 mm NaCl, 3.08 mm KCl, 11 mm glucose, 25 mm NaHCO3, 1.25 mm MgSO4, 2.52 mm CaCl2, and 1.18 mm KH2PO4, pH 7.4). The spinal cord was then transferred to the recording chamber to recover at room temperature for 1 h before recording in Ringer’s solution.

#### Isolated Whole-Cord Recordings

Electroneurogram (ENG) recordings of the (lumbar) L2 and L5 ventral roots were conducted using differential AC amplifier (A-M system, model 1700) with the band-pass filter between 300 Hz and 1 kHz. Analog signals were transferred and recorded through the Digidata 1400A board (Molecular Devices) under the control of pCLAMP10.3 (Molecular Devices). Fictive locomotor activity was induced by applying 5-hydroxytryptamine (5-HT, 8 μM) and NMDA (7–8 μM) in the Ringer’s solution.

#### Optical Stimulation of V3 Interneurons

To activate ChR2 in V3 interneurons, 488 nm fluorescent light was delivered by Colibri.2 illumination system (Zeiss) through 10x or 20 × 1.0 numerical aperture (NA) objectives mounted on an up-right microscope (Examiner, Zeiss) onto the ventral surface of the isolated spinal cord, a protocol adopted from other studies (Hägglund et al., 2013; Levine et al., 2014). This allowed us to have relative fixed focal areas. In addition, using the Colibri system, we were able to control the LED light intensity accurately. Only when we needed to compare the intensity-dependent changes, we would manually adjust the focal size of the field diaphragm and LED light intensity.

To perform unilateral stimulation, we manually adjusted the field diaphragm and LED light intensity to cover the whole half side of the spinal cord to be the largest illuminated area, and then to reduce the illuminated region between approximately one-third to a half of the spinal cord (Figure 4B) to regulate the number of V3 neurons being activated. For each cord, we set the stimuli with largest focal area and 100% light intensity as the high intensity group, then reduced focal area to medium group, and the smallest focal area less than medium group (one of third) and/or reduced light intensity to less than 50% as the low intensity group.

Continuous light stimuli with duration of 10 and 20 s were used. To use such long-lasting stimuli, we needed to make sure that they do not cause any damage of tissue and neuronal behavior. Our special studies did not find any damage produced by such stimulations in our experiments including possible effects on patch-clamp or whole cord recordings. The results of such experiments are shown in Supplementary Figure 1. Specifically, under whole cell-patch-clamp configuration, we used three consecutive 20-s light pulses to EYFP or tdTomato positive cell and showed that the firing patterns of cells were the same during each stimulation (Supplementary Figure 1A1) and their response to injected current the same before and after optical stimulation (Supplementary Figure 1A2; *n* = 22). In contrast, under the same conditions, the EYFP and tdTomato-negative neurons didn’t respond to the light stimulation (Supplementary Figure 1B1) but could generate normal spiking activity in response to injected current (Supplementary Figure 1B2; *n* = 10). To further verify that the optical stimulation did not affect neuronal activity, we applied maximum optical stimulation (100% light intensity, 10x objective) on the spinal cord of Cre negative Ai32 mice. The activity of all recorded nerves remained unchanged under and after the 20-s light pulses (Supplementary Figure 1C).

#### Whole-Cell Patch-Clamp Recordings

The experimental procedures were detailed in Borowska et al. (2013). Briefly, 300 μM slices from the spinal cord lumbar region (T13–L3) from P2-3 Sim1cre-Ai32 or Sim1^Cre/+^; tdTom; Ai32 mice were prepared in an ice-cold oxygenated sucrose solution (3.5 mm KCL, 25 mm NaHCO3, 1.2 mm KH2PO4, 1.3 mm MgSO4, 1.2 mm CaCl2, 10 mm glucose, 212.5 mm sucrose, and 2 mm MgCl2, pH 7.4) on a vibratome (Vibratome 300, Vibratome). Slices were incubated in an oxygenated Ringer’s solution (111 mm NaCl, 3.08 mm KCl, 11 mm glucose, 25 mm NaHCO3, 1.25 mm MgSO4, 2.52 mm CaCl2, and 1.18 mm KH2PO4, pH 7.4) at room temperature for >30 min for recovery before recording. EYFP fluorescence-positive, tdTom fluorescence-positive and fluorescence-negative cells were visually identified using a 40× water-immersion objective (numerical aperture, 0.8) with the aid of a DAGE-MTI IR-1000 CCD camera.

Conventional whole-cell patch-clamp recordings were made in voltage- and current-clamp modes using a MultiClamp 700B amplifier (Molecular Devices). Analog signals were filtered at 10 kHz with the Digidata 1400A board (Molecular Devices) under control of pCLAMP10.3 (Molecular Devices). Patch-clamp recording pipettes with a resistance of 5–8 MΩ were filled with solution containing 128 mm K-gluconate, 4 mm NaCl, 0.0001 mm CaCl2, 10 mm HEPES, 1 mm glucose, 5 mm Mg-ATP, and 0.3 mm GTP-Li, pH 7.2 and the fluorescent light was delivered through the 40x objective.

#### Immunohistochemistry and Confocal Image Capture

Following recording, 300 μM spinal slices from P2-3 Sim1^Cre/+^; tdTom; Ai32 mice were fixed in 4% paraformaldehyde (Electron Microscopy Science) for 10 min. Sections were then rinsed three times in 0.1% Triton X-100 (PBS-T) for 1 h total (20 min each rinse) at room temperature. Sections were then incubated in primary antibody solution for 2 days at 4°C. Primary antibody solutions consisted of 90% PBS, 10% Horse Serum (Invitrogen), and primary antibodies. Primary antibodies used were Rabbit anti DsRed (1:2000, Cat#600-401-379, Rockland) and Chicken anti GFP (1:1000, Cat#GFP-1010, Aves Labs). Following primary antibody incubation, sections were rinsed three times in PBS for 1 h total (20 min each rinse) at room temperature. Sections were then incubated in secondary antibody solution overnight at 4°C. Secondary antibody solutions consisted of 90% PBS, 10% Horse Serum (Invitrogen), Alexa Fluor 647-conjugated streptavidin (Jackson ImmunoResearch Laboratories, Inc.), and secondary antibodies. Secondary antibodies used were Fluorescein-Labelled Goat anti-Chicken IgY (1:500, Cat#F-1005, Aves Labs) and Alexa Fluor^®^ 594-conjugated Donkey Anti-Rabbit IgG (1:500, Cat# 711-585-152, Jackson ImmunoResearch Laboratories, Inc.). Lastly, sections were rinsed in PBS for 1 h total (20 min each rinse) at room temperature. Sections were then mounted on glass slides (Fisherbrand) with Dako fluorescent mounting medium and 1.5 cover slips (VWR). All images were collected on a Zeiss LSM 710 upright confocal microscope.

### Data Analysis

All recorded traces were transferred to Spike2 (Version 7.09a, Cambridge Electronic Design). The raw recordings were initially rectified and smoothed (τ = 0.05 s) using Spike2. To measure the durations of flexor and extensor bursts and the step cycle we used the smoothed signals to identify burst onset and offset times. To identify these times in noisy recordings we set a threshold that was equal to 15% of the bust amplitude (difference between maximal and minimal values) and identified intersections of the smoothed signals with the threshold. The duration of each burst was measured as a time interval between burst’s onset and offset. The step cycle duration was measured as a time interval between onset times of two consecutive bursts. The average of step cycle durations and burst durations of five consecutive cycles before the light-on and all the cycles during the light were used as a pair of data before and during light, respectively. The steps interrupted at the beginning or the end of light were excluded from the calculation.

Statistical analysis was performed in Prism7 (GraphPad Software, Inc.). Wilcoxon signed-rank test was used to compare the difference between the activity before and after the light. Linear contrasts were used to determine the relationship of the burst duration difference of activities in four nerves and step cycle difference in ipsilateral and contralateral side among three intensities. One-way ANOVA was used to determine the statistical significance among the burst duration of activity in four nerves. An α-error of *P* < 0.5 was regarded as significant. Data in the Results represent the mean ± SD.

### Computational Modeling

#### Neuron Model

All neurons were simulated in the Hodgkin–Huxley style as single-compartment models. The membrane potential, *V*, in neurons of the left and right flexor and extensor centers was described by the following differential equation

(1) $$C\times \frac{d\,V}{d\,t}=-I_{N\,a}-I_{N\,a\,P}-I_{K}-I_{L}-I_{S\,y\,n\,E}-I_{S\,y\,n\,l},$$

where *C* is the membrane capacitance and *t* is time.

In all other populations, the neuronal membrane potential was described as follows:

(2) $$C\times \frac{d\,V}{d\,t}=-I_{N\,a}-I_{K}-I_{L}-I_{S\,y\,n\,E}-I_{S\,y\,n\,l}-I_{C\,h\,R}.$$

The ionic currents in Equations (1) and (2) were described as follows:

(3) $$I_{N\,a\,P}={\bar{g}}_{N\,a}\times m_{N\,a}^{3}\times h_{N\,a}\times \left(V-E_{N\,a}\right);$$

$$I_{N\,a\,P}={\bar{g}}_{N\,a\,P}\times m_{N\,a\,P}^{3}\times h_{N\,a\,P}\times \left(V-E_{N\,a}\right);$$

$$I_{K}={\bar{g}}_{K}\times m_{K}^{4}\times \left(V-E_{K}\right);$$

$$I_{L}=g_{L}\times \left(V-E_{L}\right);$$

$$I_{C\,h\,R}=g_{C\,h\,R}\times \left(V-E_{C\,h\,R}\right),$$

where *I*_*Na*_ is the fast Na^+^ current with maximal conductance ${\bar{g}}_{N\,a}$; *I*_*NaP*_ is the persistent (slowly inactivating) Na^+^ current with maximal conductance ${\bar{g}}_{N\,a\,P}$ (present only in RG neurons); *I*_*K*_ is the delayed-rectifier K^+^ current with maximal conductance ${\bar{g}}_{K}$; *I*_*L*_ is the leakage current with constant conductance *g*_*L*_; *I*_*ChR*_ is the channelrhodopsin current with the conductance *g*_*ChR*_ (present only in V3 neurons). *E*_*Na*_, *E*_*K*_, *E*_*L*_, and *E*_*ChR*_ are the reversal potentials for Na^+^, K^+^, leakage, and channelrhodopsin currents, respectively; variables *m* and *h* with indexes indicating ionic currents are the activation and inactivation variables of the corresponding ionic channels.

Activation *m* and inactivation *h* of voltage-dependent ionic channels (e.g., Na, NaP, and K) in Equation (3) were described by the following differential equations:

(4) $$\tau_{m\,i}\,\left(V\right)\times \frac{d}{d\,t}\,m_{i}=m_{\infty \,i}\,\left(V\right)-m_{i};$$

$$\tau_{h\,i}\,\left(V\right)\times \frac{d}{d\,t}\,h_{i}=h_{\infty \,i}\,\left(V\right)-h_{i},$$

where *m*_∞_*_*i*_*(*V*) and *h*_∞_*_*i*_*(*V*) define the voltage-dependent steady-state activation and inactivation of the channel *i*, respectively, and *τ_*mi*_*(*V*) and *τ_*hi*_*(*V*) define the corresponding time constants. Activation of the sodium channels is considered to be instantaneous. The expressions for channel kinetics in Equation (4) are described as follows:

(5) $$m_{\infty \,N\,a}\,\left(V\right)={\left(1+\exp\left(-\left(V+34\right)/7.8\right)\right)}^{-1};$$

$$\tau_{m\,N\,a}=0;$$

$$h_{\infty \,N\,a}\,\left(V\right)={\left(1+\exp\left(\left(V+55\right)/7\right)\right)}^{-1};$$

$$\tau_{h\,N\,a}\,\left(V\right)=20/\left(\exp\left(\left(V+50\right)/15\right)\right)+\exp\left(-\left(V+50\right)/16\right);$$

$$m_{\infty \,N\,a\,P}\,\left(V\right)={\left(1+\exp\left(-\left(V+47.1\right)/3.1\right)\right)}^{-1};$$

$$\tau_{m\,N\,a\,P}=0;$$

$$h_{\infty \,N\,a\,P}\left(V\right)=(1+\exp{\left(\left(V+60/6.8\right)\right)}^{-1};$$

$$\tau_{h\,N\,a\,p}\,\left(V\right)=8000/\cosh\left(\left(V+60\right)/13.6\right);$$

$$m_{\infty \,K}\,\left(V\right)={\left(1+\exp\left(-\left(V+28\right)/4\right)\right)}^{-1};$$

$$\tau_{m\,K}\,\left(V\right)=3.5/\cosh\left(\left(V+40\right)/40\right);$$

$$h_{K}=1.$$

The maximal conductances for ionic currents and the leak reversal potentials, *E*_*L*_, for different populations are given in Table 1.

The synaptic excitatory (*I*_*SynE*_ with conductance *g*_*SynE*_ and reversal potential *E*_*SynE*_) and inhibitory (*I*_*SynI*_ with conductance *g*_*SynI*_ and reversal potential *E*_*SynI*_) currents were described as follows:

(6) $$I_{S\,y\,n\,E}=g_{S\,y\,n\,E}\times \left(V-E_{S\,y\,n\,E}\right);$$

$$I_{S\,y\,n\,I}=g_{S\,y\,n\,I}\times \left(V-E_{S\,y\,n\,I}\right).$$

where *g*_*SynE*_ and *g*_*Synl*_ are equal to zero at rest and are activated by the excitatory or inhibitory inputs, respectively:

(7) $$g_{S\,y\,n\,E_{i}}\,\left(t\right)={\bar{g}}_{E}\times \sum_{j}S\,\left\{w_{j\,i}\right\}\times \sum_{t_{k\,j}<t}\exp\left(-\left(t-t_{k\,j}\right)/\tau_{S\,y\,n\,E}\right);$$

$$g_{S\,y\,n\,l\,i}\,\left(t\right)={\bar{g}}_{I}\times \sum_{j}S\,\left\{-w_{j\,i}\right\}\times \sum_{t_{k\,j}<t}\exp\left(-\left(t-t_{k\,j}\right)/\tau_{S\,y\,n\,l}\right),$$

where *S*{*x*} = *x*, if *x* ≥ 0, and 0 if *x* < 0. Each spike arriving to neuron *i* in a target population from neuron *j* in a source population at time *t*_*kj*_ increases the excitatory synaptic conductance by ${\bar{g}}_{E}\times w_{j\,i}$ if the synaptic weight *w*_*ji*_ > 0, or increases the inhibitory synaptic conductance by $-{\bar{g}}_{I}\times w_{j\,i}$ if the synaptic weight *w*_*ji*_ < 0. ${\bar{g}}_{E}$ and ${\bar{g}}_{I}$ define an increase in the excitatory or inhibitory synaptic conductance, respectively, produced by one arriving spike at | *w*_*ji*_| = 1. τ_*SynE*_ and τ_*SynI*_ are the decay time constants for *g*_*SynE*_ and *g*_*SynI*_, respectively.

The following general neuronal parameters were assigned: *C* = 1 μF ⋅ cm^–2^; *E*_*Na*_ = 55 mV; *E*_*K*_ = - 80 mV; *E*_*ChR*_ = −10 mV; *E*_*SynE*_ = -10 mV; *E*_*SynI*_ = -70 mV; ${\bar{g}}_{E}$ = ${\bar{g}}_{I}$ = 0.05 mS/cm^2^; τ_*SynE*_ = τ_*SynI*_ = 5 ms.

#### Neuron Populations

Each neuron population in the model contained 50–200 neurons. The numbers of neurons in each population are shown in Table 1.

Random synaptic connections between the neurons of interacting populations were assigned prior to each simulation based on probability of connection, *p*, so that, if a population *A* was assigned to receive an excitatory (or inhibitory) input from a population *B*, then each neuron in population *A* would get the corresponding synaptic input from each neuron in population *B* with the probability *p*{*A*, *B*}. If *p*{*A*, *B*} < 1, a random number generator was used to define the existence of each synaptic connection; otherwise [if *p*{*A*, *B*} = 1] each neuron in population *A* received synaptic input from each neuron of population *B.* Values of synaptic weights (*w*_*ji*_) were set using random generator and were based on average values of these weights $\bar{w}$ and variances, which were defined as 5% of $\bar{w}$ for excitatory connections ($\bar{w}$ > 0) and 10% of $\bar{w}$ for inhibitory connections ($\bar{w}$ < 0). The average weights and probabilities of connections are specified in Table 2.

Heterogeneity of neurons within each population was provided by random distributions of the base values of the mean leakage reversal potentials ${\bar{E}}_{L\,i\,0}$ (see mean values ± SD for each *i*-th population in Table 1) and initial conditions for the values of membrane potential and channel kinetics variables. The base values of ${\bar{E}}_{L\,i\,0}$ and all initial conditions were assigned prior to simulations from their defined average values and variances using a random number generator, and a settling period of 10–200 s was allowed in each simulation.

#### Simulations of Changes in the Locomotor Frequency by Neuroactive Drugs and Application of Photostimulation

In the model, the frequency of rhythmic oscillations depended on the parameter α, that defined the level of average neuronal excitation in each population *i* (Shevtsova et al., 2015):

${\bar{E}}_{L\,i}={\bar{E}}_{L\,i\,0}\times \left(1-\alpha \right)$ where ${\bar{E}}_{L\,i\,0}$ represents the base value of mean leakage reversal potential in the population at α = 0 (see Table 1).

To simulate the effect of photostimulation, we selectively activated the V3 neurons either bi- or unilaterally by increasing the channelrhodopsin current conductance (*g*_*ChR*_), which was set to 0 in control conditions.

To estimate changes in model behavior after increase of the channelrhodopsin current conductance (*g*_*ChR*_), we used two methods. In the first method, simulations were run at the fixed value of *g*_*ChR*_ (Figures 8, 10, 11B2). The values of *g*_*ChR*_ for these particular simulations are indicated in the corresponding figure legends. This method was also used to estimate the robustness of the model and simulate variability of experimental condition and individual spinal cords (Figures 9B,C, 12). To do this, we performed a series of simulations for both experimental conditions (bilateral and unilateral activation of V3 neurons) when parameters α, defining the level of average neuronal excitation in each population, and *g*_*ChR*_, characterizing intensity of photostimulation, were randomly chosen in the range [0.01, 0.06] for α and [0.18, 0.4] for *g*_*ChR*_. For each pair (α, *g*_*ChR*_) a simulation was run and average values for flexor and extensor burst durations and oscillation period were calculated and compared with control condition when the model behavior was estimated with the same α and *g*_*ChR*_ = 0.

In the second method, the integrated population activities were computed continuously during slow-ramp increase of *g*_*ChR*_. This method was used to study the effect of stimulation intensity on model behavior in more detail (see Figures 9A, 11A1,B1). The value of *g*_*ChR*_ was slowly increased from 0 to 0.4 (0.01 during 100 s of simulated time). For each locomotor cycle durations of flexion and extension phases or number of ipsilateral flexor bursts were calculated and plotted against the parameter *g*_*ChR*_.

#### Computer Simulations

All simulations were performed using the custom neural simulation package NSM 2.5.11^1^ developed at Drexel University by S. N. Markin, I. A. Rybak, and N. A. Shevtsova. This simulation package was previously used for the development of several spinal cord models (Rybak et al., 2006a, b, 2013; McCrea and Rybak, 2007, 2008; Zhong et al., 2012; Shevtsova et al., 2015; Shevtsova and Rybak, 2016). Differential equations were solved using the exponential Euler integration method with a step size of 0.1 ms. Simulation results were saved as ASCI files containing time moments of spikes for RG populations. Model configuration files to create the simulations presented in the paper are available at https://github.com/RybakLab/nsm/tree/master/models/Danner-2019-V3.

#### Data Analysis in Computer Simulations

The results of simulations were processed by custom Matlab scripts (The Mathworks, Inc., Matlab 2019a). To assess the model behavior, the averaged integrated activities of flexor and extensor centers (average number of spikes per neuron) were used to calculate the flexor and extensor burst durations and oscillation period. The timing of onsets and offsets of flexor and extensor bursts was determined at a threshold level equal to 10–25% of the average difference between maximal and minimal burst amplitude for particular population in the current simulation. The locomotor period was defined as the duration between two consecutive onsets of the extensor centers. Duration of individual simulations depended on the value of parameter α, and to robustly estimate average value of burst duration and oscillation period in the first method, the first 10–20 transitional cycles were omitted to allow stabilization of model variables, and the values of model parameters were averaged for 10–20 consecutive cycles. In the second method, to validate our results we selectively run additional simulation with a slower ramp increase (0.01 during 500 s of simulated time) or with a fixed value of parameter in proximity of bifurcation points.

## Data Availability Statement

The datasets generated for this study are available on request to the corresponding author. The simulation software (NSM) and model configuration files are publically available at https://github.com/RybakLab/nsm.

## Ethics Statement

The animal study was reviewed and approved by the University Committee on Laboratory Animals at Dalhousie University.

## Author Contributions

SD, HZ, NS, IR, and YZ: conceptualization, methodology, and writing (original draft preparation, review, and editing). YZ, HZ, JB-F, and DD-G: experiments. SD and NS: modeling. SD, HZ, and NS: formal analysis and software. HZ and SD: data curation. SD, HZ, and NS: visualization. IR and YZ: supervision, project administration, and funding acquisition.

## Conflict of Interest

The authors declare that the research was conducted in the absence of any commercial or financial relationships that could be construed as a potential conflict of interest.

## Footnotes

1 https://github.com/RybakLab/nsm

## Supplementary Material

FIGURE S1 Long-pulse photoactivation did not damage cells. Representative traces of whole cell patch-clamp recordings of ChR2-EYFP positive **(A1)** and negative **(B1)** neurons receiving 3 consecutive 20-s 488 fluorescent light-pulses and representative traces of whole cell patch-clamp recordings of ChR2-EYFP positive **(A2)** and negative **(B2)** neurons injected with 1-s 50 pA depolarization current before (left) and after (right) the 3 light stimulations. **(C)** A lack of response of Ai32 cord to the light.
